# An Orally Bioavailable (Mice) Prodrug of Glutathione

**DOI:** 10.3390/antiox10060939

**Published:** 2021-06-10

**Authors:** Daune L. Crankshaw, Jacquie E. Briggs, Robert Vince, Herbert T. Nagasawa

**Affiliations:** 1 Center for Drug Design, University of Minnesota, Minneapolis, MN 55455, USA; cranksdl@umn.edu (D.L.C.); brigg006@umn.edu (J.E.B.); vince001@umn.edu (R.V.); 2 Department of Veterans Affairs Medical Center, One Veterans Drive, Minneapolis, MN 55417, USA

**Keywords:** prodrug, glutathione, orally, bioavailable

## Abstract

L-Cysteine-glutathione mixed disulfide (CySSG), a prodrug of glutathione (GSH), was found to be orally bioavailable in mice, and protected mice against a toxic dose of acetaminophen. If oral bioavailability can also be demonstrated in humans, a wide range of applicability for CySSG can be envisioned.

## 1. Introduction

The very important role played by endogenous glutathione (GSH), known as the ‘master antioxidant’, is well known and need not be further elaborated. However, not so well known is the observation that the first enzyme in the two-step biosynthesis pathway for GSH is compromised as we get older, especially beyond the age of 60 [1,2]. Taking supplemental GSH orally is not an option, since oral GSH is rapidly degraded by the enzyme, gamma-glutamyl transpeptidase [2]. An orally bioavailable GSH has not yet been described in the literature; therefore, we now present an orally biooavailable GSH in the form of its prodrug.

In an earlier publication [3] we reported that the mixed disulfide of L-cysteine and GSH, viz., CySSG (Sis-Gee), protected mice against acetaminophen-induced hepatotoxicity when administered intraperitoneally. Here, we show that CySSG, which is naturally found in human blood [4] and is a prodrug of GSH, also protected mice against acetaminophen overdose when administered by oral gavage. On net reduction of its disulfide bond in vivo in a thiol-disulfide exchange reaction [5], CySSG releases not only GSH but also an equivalent of L-cysteine, the rate-limiting amino acid required for the de novo biosynthesis of GSH.

## 2. Materials and Methods

**Figure 1:**
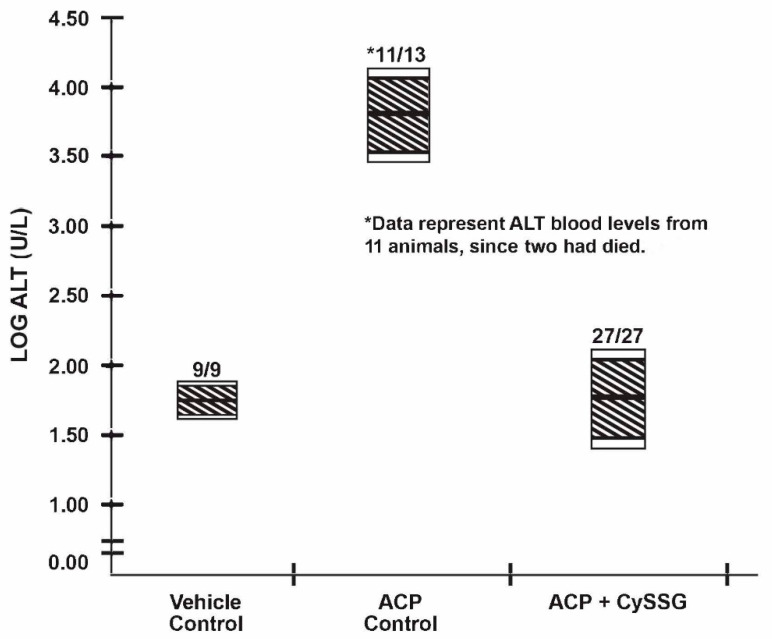
Alanine transaminase (ALT) levels of mice treated orally with CySSG.

Fasted (12 h) male, Swiss-Webster mice weighing 25–35 g (total numbers indicated in Figure 1), purchased from Harlan-Sprague Dawley (Indianapolis, IN), were administered intraperitoneally with 2.45 mmol/kg of ACP dissolved in saline. The vehicle control mice received only saline. For mice receiving the protective agent, this was followed 50 min. later with 2.50 mmol/kg of CySSG orally by gavage. The CySSG was dissolved in a minimum amount of diluted HCl, the solution neutralized with diluted NaOH and made to requisite volume with isotonic saline. For the CySSG group, three separate experiments with n’s of 9 were combined. At 24 h post-ACP, mice were anesthetized with Ketamine/Xylazine (ip). The degree of anesthesia was determined by non-response to pinch, and blood was withdrawn by cardiac puncture for determination of plasma ALT levels using a commercial assay kit from Sigma-Aldrich. The data were plotted in a manner that allowed ready comparison between data by presenting them in a logarithmic scale [5] to accommodate the very large differences in numbers, e.g., log 2 = 100; log 4 = 10,000. This condensed the chart to reflect the serum ALT levels of the mice at 24 h post acetaminophen with and w/o treatment with CySSG, a quantitative indication of hepatic damage.

The use of mice for this project, “Oral Glutathione Animal Protocol”, was approved by the University of Minnesota Animal Care and Use Committee (U of MN IACUC 70806).

## 3. Results

Figure 1 describes the data which show the efficacy of orally administered CySSG in protecting mice against a toxic dose of acetaminophen. The hatched areas of the vertical bars reflect the 99% confidence interval, while the unhatched (clear) extension of the bars reflect the 95% confidence interval of the log-transformed ALT values pursuant to a previous study [6]. Thus, a horizontal intersect of the hatched areas of any two data bars (use of a straightedge is recommended) indicates that the data are identical at the 99% level of confidence; whereas a horizontal intersect encompassing the unhatched areas indicates that the data are identical at the 95% confidence interval.

It can be seen that the ACP Control mice had some toxic deaths (2 of 13), as well as severe elevations in blood ALT here levels indicated by the large log values, whereas mice given identical ACP doses plus oral CySSG survived with essentially normalized blood ALT levels. The numbers of mice in the Vehicle Control and ACP Control Groups were intentionally minimized to reduce animal use. This did not affect the results observed.

## 4. Discussion

Oral administration of CySSG to mice treated with a toxic dose of acetaminophen fully protected the animals, as indicated by their serum ALT levels which were not different from the vehicle controls (Figure 1). Thus, CySSG has now been shown in this mouse model to be orally bioavailable for the delivery of GSH. While mice and rat data are generally transferable to humans, it is incumbent that studies be conducted with CySSG in humans as soon as possible by clinician investigators relative to its oral bioavailability. GSH taken orally by humans is degraded by the enzyme, gamma-glutamyl transpeptidase [2], and is known not to be bioavailable [7]. In contrast, the mechanism that releases GSH from CySSG intracellularly is via a thiol-disulfide exchange reaction [5], and was shown here to be bioavailable in mice.

As alluded to earlier, the first enzyme in the two-step de novo biosynthetic pathway for GSH is greatly compromised in people older than 60 years [1,2]. This detriment in our older populaticn creates a dilemma, since taking GSH orally does not solve this problem, and requires a bioavailable form of GSH itself. We have now provided this as CySSG, a bioavailable form of GSH, which should be of great benefit to our older generation.

Moreover, an orally bioavailable GSH is of particular relevance in this era of worldwide COVID-19 pandemic. For hospitalized COVID-19 infected patients, intravenous GSH is being recommended [8,9]. to relieve the severity of this dreaded disease, and it would be of interest to compare oral CySSG with intravenously administered GSH. Indeed, an orally bioavailable CySSG should be recommended for all ambulatory COVID-infected patients, as well as for all individuals nearing or past the age of 60.

## 5. Conclusions

Since GSH is known NOT to be orally bioavailable, we have developed a bioavailable form of GSH in the form of Its prodrug, cysteine-glutathione mixed disulfide, viz., CySSG. When administered orally to mice, CySSG protected the mice from a toxic dose of acetaminophen (Figure 1), a well-known hepatotoxic agent. An orally bioavailable CySSG should be applicable to variety of situations mentioned above.

## 6. Patents

The Dept. of Veterans Affairs (DVA), Washington DC, has assigned the patent rights for CySSG to a commercial entity for further development.

## Data Availability

The data presented in this study are available on request from the corresponding author.

## Abbreviations

ACP	Acetaminophen
ALT	Alanine transaminase
CySSG	Cysteine-glutathione mixed disulfide
GSH	Glutathione
